# Patients’ Expectations and Experiences With a Mental Health–Focused Supportive Text Messaging Program: Mixed Methods Evaluation

**DOI:** 10.2196/33438

**Published:** 2022-01-11

**Authors:** Reham Shalaby, Wesley Vuong, Ejemai Eboreime, Shireen Surood, Andrew J Greenshaw, Vincent Israel Opoku Agyapong

**Affiliations:** 1 Department of Psychiatry University of Alberta Edmonton, AB Canada; 2 Addiction and Mental Health Alberta Health services Edmonton, AB Canada

**Keywords:** supportive text messages, patients’ experience, mental health, mixed methods

## Abstract

**Background:**

Web-based services are an economical and easily scalable means of support that uses existing technology. Text4Support is a supportive, complementary text messaging service that supports people with different mental health conditions after they are discharged from inpatient psychiatric care.

**Objective:**

In this study, we aim to assess user satisfaction with the Text4Support service to gain a better understanding of subscribers’ experiences.

**Methods:**

This was a mixed methods study using secondary data from a pilot observational controlled trial. The trial included 181 patients discharged from acute psychiatric care and distributed into 4 randomized groups. Out of the 4 study groups in the initial study, 2 groups who received supportive text messages (89/181, 49.2% of patients), either alone or alongside a peer support worker, were included. Thematic and descriptive analyses were also performed. Differences in feedback based on sex at birth and primary diagnosis were determined using univariate analysis. The study was registered with ClinicalTrials.gov (trial registration number: NCT03404882).

**Results:**

Out of 89 participants, 36 (40%) completed the follow-up survey. The principal findings were that Text4Support was well perceived with a high satisfaction rate either regarding the feedback of the messages or their perceived impact. Meanwhile, there was no statistically significant difference between satisfactory items based on the subscriber’s sex at birth or primary diagnosis. The patients’ initial expectations were either neutral or positive in relation to the expected nature or the impact of the text messages received on their mental well-being. In addition, the subscribers were satisfied with the frequency of the messages, which were received once daily for 6 consecutive months. The participants recommended more personalized messages or mutual interaction with health care personnel.

**Conclusions:**

Text4Support was generally well perceived by patients after hospital discharge, regardless of their sex at birth or mental health diagnosis. Further personalization and interactive platforms were recommended by participants that may need to be considered when designing similar future services.

## Introduction

### Background

Recently, there has been rapid adoption of computer- and web-based services in health care systems. These services are often highly accessible, remotely delivered, cost-effective, and easy to use [1-3]. These characteristics make computer- and web-based services appealing and attractive to both health care providers and patients.

Wireless and mobile technologies needed to deliver computer- and web-based services have been rapidly expanding. In 2019, there were >8 billion mobile phone subscriptions and >4 billion wireless internet users worldwide [4]. Given this vast reach, the use of these technologies may be beneficial in community mental health where accessibility concerns, service gaps, and high cost of services are often reported [5].

With mobile technologies, text messaging services are increasingly used to serve nontraditional health care service functions across different health concerns. For instance, texting services have been used as medical appointment reminders [6] and to help encourage patients to adhere to medication use [7]. There are approximately 400 mobile phone apps and service programs aimed at helping adults and pregnant women with smoking cessation and improving health beliefs and attitudes for new mothers (eg, Text2Quit, a text messaging program for smoking cessation, and Quit4Baby, a smoking cessation text messaging program for pregnant smokers, both developed and operated by Voxiva Inc) [8-11]. Similarly, Text4Mood and Text4Hope are examples of mobile texting services for mental health. Both programs aimed to help support individuals living with mood disorders and to provide mental health support to the public during the COVID-19 pandemic [2,12].

### Objectives

Text4Support is a service offered by Alberta Health Services, a health authority in the province of Alberta, Canada. This complementary service began in 2018 to support people living with different mental health conditions [13]. Psychiatrists, psychologists, and mental health therapists developed cognitive behavioral therapy (CBT)–based text messaging content. CBT focuses on helping individuals manage their concerns, primarily by targeting negative beliefs and coping behaviors [14].

The purpose of this initiative is to assess user satisfaction and better understand the subscribers’ experience with Text4Support. The assessment of user satisfaction can lead to better client retention and clinical outcomes [15]. A recent study reported that a 7.2% reduction in the frequency of reporting *at least good overall satisfaction* was associated with a 1% increase in hospital bed occupancy [15]. Similarly, texting and web-based services are widely accepted by individuals who perceive these services as supportive and acceptable [1,16]. Overall, examining patients’ expectations and experiences can help allocate resources, and positive expectations are highly linked to the patient’s clinical outcome [17,18].

A recent evaluation of Text4Support indicates that the program is effective and accepted by individuals seeking access to outpatient mental health services in the Edmonton Zone. A large proportion of these subscribers reported always or often reading of the messages (25/26, 96%), and after receiving the service for 6 months, the majority agreed that the text messages (TxMs) were to the point (18/24, 75%), supportive (22/24, 92%), and positive (22/24, 92%) [19]. In a similar study, the authors reported higher satisfaction with the texting service among female participants, who reported an improvement in coping with different stresses related to the COVID-19 pandemic along with an improvement in their quality of life, compared with their male counterparts [3]. In addition, the literature reported high satisfaction related to different mental health conditions, such as psychotic disorder and depression [1,3,19]. However, the results based on sex at birth or diagnosis were not significant for any of the assessed items. There is also a dearth of research focusing on satisfaction and acceptability among patients based on their diagnosis and in a comparative fashion. This study examines such differences and aims to provide an additional layer of evidence to the field.

This study focuses on the experience of inpatients who are enrolled in Text4Support after their discharge from the mental health units in Edmonton, Alberta. Subscribers are patients with different mental health disorders who received daily TxMs for 6 months.

## Methods

### Research Goals

Research goals included the following: (1) to explore and evaluate the experiences of patients who had been recently discharged from acute care mental health units regarding the supportive TxM service (Text4Support), from which they received daily messages for a 6-month period, and (2) to explore any differences in satisfaction based on sex at birth or the primary diagnosis of subscribers.

### Study Design

This was a mixed methods study (qualitative and quantitative) with data gathered using patients’ key informant interviews and a web-based survey. Quantitative and qualitative methods were guided by the Checklist for Reporting of Survey Studies [20] and the consolidated criteria for reporting qualitative research [21], respectively.

### Setting and Study Participants

Data were collected from the subscribers who joined the Text4Support program as part of a controlled observational clinical trial [13]; subscribers received daily supportive TxMs for 6 weeks on their registered mobile phone numbers. The detailed recruitment process is described elsewhere [22] and is briefly highlighted here.

The study was conducted at 5 acute psychiatric care units in Edmonton, Alberta, Canada. Patients were invited to participate in the study from June 2019 to February 2020 before their hospital discharge. The selection criteria for the program were as follows: patients who were hospitalized and imminent to be discharged; patients aged 18-65 years; patients who had a mental health condition other than substance use disorder (eg, mood or psychotic disorder); patients who were able to provide written consent; and patients who had a mobile phone capable of receiving TxMs.

The research team applied a random allocation thereafter to assign the patients to four study arms: (1) peer support worker (PSW) only, (2) TxM only, (3) PSW plus TxM condition, and (4) treatment as usual.

For the purpose of this study, we focused on the 2 study arms who received TxM services (TxM only and PSW plus TxM). The patients received daily TxMs for 6 months, and we examined the midpoint experience after 6 weeks of receiving the TxM service.

### Text4Support Background and Data Collection

Text4Support is a texting mental health service conceived and designed by a group of psychiatrists, psychologists, and mental health therapists based on the concepts of CBT [23]:

- A bank of messages was generated and included eight different streams of messages tailored for the following mental health conditions: depression, anxiety, psychotic disorders, bipolar disorder, general well-being, anxiety, substance use, personality disorder, and adjustment disorder. About 80% (144/180) of the messages shared a similar content, and 20% (36/180) of the messages were mental health condition–specific.
- The consenting participants provided the research team with their mobile phone numbers. This number was added to a texting delivery platform so that clients received daily messages that catered to their primary mental health concern.
- Patients received automated messages with content aligned with their current areas of diagnosis or concern every day at noon (MST) for 6 months.
- A midpoint web-based satisfaction survey was sent to patients 6 weeks after enrollment in the service.

### Examples of the Messages

Following are examples of general supportive messages:

Think of your recovery as an opportunity to find new solutions in your life.

Remember that the past is gone and what you do is what really matters for depression.

Following are examples of messages sent for depression:

Monitor your mood from on scale from 1-10 with 1 being lowest and 10 highest. Make a note of activities that improve your mood.

When you notice a change in your mood, ask yourself what went through your mind. Did you notice a thought or an image?

Following are examples of messages sent for anxiety:

When you notice an increase in anxiety, pay attention to what triggered it - an interaction, a situation, a memory, a thought, etc.

Make a list of what you’re avoiding. Rate how anxious each thing makes you. Do what makes you least anxious and work your way up.

### Quantitative Data

The (midpoint) satisfaction survey included an adopted version of the Text4Mood user satisfaction survey [2]. The survey took 5-10 minutes to complete, and receiving supportive TxMs was not contingent on survey completion.

The survey questions were formulated based on the objectives of the study and available evidence from peer-reviewed literature [2]. The survey consisted of predominately Likert scale responses that evaluated sociodemographic and clinical characteristics, subscribers’ responses to and perceptions of the supportive TxMs, and the impact of the program on subscribers’ mental well-being. Data were collected between August 2019 and February 2020. The instrument was not validated but was adopted from surveys used in previous text messaging programs [2,3,19]. Participants entered their mobile phone numbers as their unique study ID number, which prevented multiple participation in the study.

### Qualitative Data

Of the 15 randomly selected participants who belonged to the text messaging group and contacted via TxMs, 7 (47%) accepted to participate in a key informant interview via telephone (Table 1). The interviews lasted between 30 and 45 minutes and were conducted by EE with experience in qualitative research. The interviews were recorded and subsequently transcribed verbatim. The interview questions (Multimedia Appendix 1) explored expectations, experiences, anticipated receptivity, and the impact of the daily supportive TxMs received for 6 months from the perspective of the patients who were recently discharged from acute care mental health units in hospitals in Edmonton, Alberta, Canada. Data were collected between August 2020 and October 2020.

**Table 1:** Demographic and clinical characteristics of the respondents to the qualitative assessment.

Participant	Age (years)	Sex	Mental health condition or diagnoses
P1	37	Female	Depression and anxiety disorder
P2	57	Female	Depression and anxiety disorder
P3	62	Female	Bipolar disorder
P4	42	Female	Depression and anxiety disorder
P5	57	Male	Bipolar disorder
P6	47	Female	Bipolar disorder
P7	52	Male	Depression and anxiety disorder

### Data Analysis

#### Quantitative Data

Data were analyzed using SPSS Statistics for Windows (version 26; IBM Corporation) [24]. The demographic characteristics were summarized as raw numbers and percentages. Likert scale satisfaction responses to various aspects of the Text4Support service were summarized as frequency counts of response categories and percentages.

We were interested in studying the feedback and satisfaction of the different participant groups. Thus, we examined each of the 25 questions in the satisfaction survey against participants’ sex at birth and admitting diagnosis using chi-square analysis and Fisher exact test with a 2-tailed probability of significance, *P*≤.05. There was no imputation for missing data, and the results were based on the completed survey responses.

#### Qualitative Data

Participants’ answers to the interview questions were transcribed and analyzed thematically using NVivo 12 (QSR International). Both inductive and deductive approaches were used in the analysis. First, structural coding was used to generate the initial codes in line with specific research questions. Thereafter, pattern coding, which allows the identification of explanatory or inferential codes, was applied to the initial codes to identify patterns or emerging themes and subthemes across the data set. Each individual theme and subtheme were further examined for *fit* against the collated extracts for each theme, subtheme, and the overall data set. The final sets of themes and subthemes were reported alongside verbatim quotes. Owing to the nature of the patients’ ill health, repeat interviews, feedback on transcripts, and analysis by participants were not sought to minimize the risk of psychological distress.

### Ethics Approval

The study was approved by the Health Ethics Research Board of the University of Alberta (reference number: Pro00078427) and received operational approval from Alberta Health Services, the regional health authority. Written informed consent was obtained from all the patients. The information sheet contains the details of the researchers and the study objectives. The study was registered with ClinicalTrials.gov (trial registration number: NCT03404882). In relation to the design change to a controlled observational study the amendments to the study protocol [13] are now reflected in a revised registered trial protocol for NCT0340488.

## Results

### Quantitative Data

Of the 89 patients allocated to the texting service, we received complete surveys from 36 (40%) participants.

Table 2 displays subscribers’ demographic characteristics, indicating that most respondents (27/36, 75%) were women, aged between 50 and 65 years (10/36, 28%), White (32/36, 89%), reported completion of postsecondary education (27/36, 77%), were unemployed (15/36, 42%), and were divorced, separated, or widowed (14/36, 39%). Most respondents (22/36, 61%) had depression or anxiety.

**Table 2:** Demographic and clinical characteristics of study participants.

Characteristics			Values, n (%)
**Sex at birth (n=36)**			
	Male	9 (25)	
	Female	27 (75)	
**Age groups (years; n=36)**			
	18-30	4 (11)	
	31-40	6 (17)	
	41-50	9 (25)	
	51-65	7 (19)	
	>65	10 (28)	
**Ethnicity (n=36)**			
	Indigenous	1 (3)	
	White	32 (89)	
	Asian	3 (8)	
**Education level (n=35)**			
	Less than high school	3 (9)	
	High school degree or equivalent	5 (14)	
	Above high school education	27 (77)	
**Employment status (n=36)**			
	Employed	13 (36)	
	Unemployed	15 (42)	
	Other	8 (22)	
**Relationship (n=36)**			
	Married, common law, or in a relationship	9 (25)	
	Single	13 (36)	
	Divorced, separated, or widowed	14 (39)	
**Admitting diagnosis (n=36)**			
	Depression or anxiety	22 (61)	
	Bipolar disorder	12 (33)	
	Psychotic disorder	2 (6)	

Table 3 illustrates the subscribers’ opinions about the Text4Support messages after receiving 6 weeks of daily TxMs. The data indicate that most of the respondents always or mostly found the TxMs positive (34/36, 95%), affirmative (34/35, 97%), and clear (34/35, 97%). Similarly, 88% (30/34) of the respondents indicated that the messages were always or often relevant.

Most participants reported that they felt supported when receiving the TxMs (30/36, 83%), always read the messages (31/36, 86%), and always understood them (28/32, 88%). Generally, most participants were satisfied with the TxMs (28/34, 82%) and indicated their preference to receive the TxMs once per day (21/34, 62%).

Table 4 data show that slightly more than 3 in 4 respondents indicated that they either read and reflected on the TxMs or took positive or beneficial actions after reading the messages (26/34, 76%). No subscribers indicated that they read the messages and took a negative or harmful action. In addition, Table 4 shows the subscribers’ level of agreement regarding the benefits of Text4Support and the perceived impact of the messages after receiving daily messages for 6 weeks. The results indicate that 4 in 5 respondents (28/35, 80%) reported that the TxMs helped them feel connected to a support system.

**Table 3:** Participants’ feedback about Text4Support after 6 weeks of intervention.

Feedback			Values, n (%)
**When you received the daily messages, how do they make you feel? (n=36)**			
	Supported	30 (83)	
	Indifferent	4 (11)	
	Annoyed	2 (6)	
**How often did you read the messages? (n=36)**			
	Always	31 (86)	
	Mostly	4 (11)	
	Rarely	1 (3)	
**How often did you understand the messages? (n=32)**			
	Always	28 (87)	
	Mostly	4 (13)	
	Rarely	0 (0)	
**Did you find the Text4Support messages to be positive? (n=36)**			
	Always	19 (53)	
	Mostly	15 (42)	
	Sometimes	2 (5)	
**Did you find the Text4Support messages to be supportive? (n=35)**			
	Always	22 (63)	
	Mostly	12 (34)	
	Sometimes	1 (3)	
**Did you find the Text4Support messages to be clear? (n=35)**			
	Always	23 (66)	
	Mostly	11 (31)	
	Sometimes	1 (3)	
**Did you find the Text4Support messages to be relevant? (n=34)**			
	Always	12 (35)	
	Mostly	18 (53)	
	Sometimes	4 (13)	
**How satisfied were you with the frequency of the text messages? (n=34)**			
	Satisfied	28 (82)	
	Neither satisfied nor dissatisfied	5 (15)	
	Dissatisfied	1 (3)	
**Ideally, how often would you prefer to receive supportive text messages? (n=34)**			
	Once daily	21 (62)	
	Twice daily	9 (26)	
	Once every other day	1 (3)	
	Once weekly	3 (9)	

**Table 4:** Perceived impact of receiving daily messages for 6 weeks.

Perceived impact of daily messages from Text4Support			Values, n (%)
**When you received the texts, what happened next? (n=34)**			
	Read text and took a positive or beneficial action	8 (23)	
	Read text and reflected on the messages	18 (53)	
	Read the text and took no action	7 (21)	
	Read text and took a negative or harmful action	0 (0)	
	Did not read the text	1 (3)	
**The daily messages from Text4Support helps me to cope with stress (n=35)**			
	Agree	22 (63)	
	Neutral	10 (28)	
	Disagree	3 (9)	
**The daily messages from Text4Support helps me to cope with loneliness (n=35)**			
	Agree	23 (66)	
	Neutral	6 (17)	
	Disagree	6 (17)	
**The daily messages from Text4Support helps me to manage suicidal thoughts (n=34)**			
	Agree	12 (35)	
	Neutral	16 (47)	
	Disagree	6 (18)	
**The daily messages from Text4Support helps me to monitor my mood (n=36)**			
	Agree	21 (58)	
	Neutral	10 (28)	
	Disagree	5 (14)	
**The daily messages from Text4Support helps me to remember my goals (n=35)**			
	Agree	25 (71)	
	Neutral	6 (17)	
	Disagree	4 (11)	
**The daily messages from Text4Support helps me feel connected to a support system (n=35)**			
	Agree	28 (80)	
	Neutral	5 (14)	
	Disagree	2 (6)	
**The daily messages from Text4Support helps me feel hopeful I can manage issues in my life (n=34)**			
	Agree	22 (65)	
	Neutral	9 (26)	
	Disagree	3 (9)	
**The daily messages from Text4Support helps me know where to get help for depression or anxiety (n=34)**			
	Agree	16 (47)	
	Neutral	12 (35)	
	Disagree	6 (18)	
**The daily messages from Text4Support helps me feel that I could be the one in charge of managing depression or anxiety (n=35)**			
	Agree	18 (51)	
	Neutral	13 (37)	
	Disagree	4 (11)	
**The daily messages from Text4Support helps me feel like I know how to stay on track when life or everyday stressors come up (n=35)**			
	Agree	23 (66)	
	Neutral	7 (20)	
	Disagree	5 (14)	
**The daily messages from Text4Support helps me feel like I am making a change (n=35)**			
	Agree	25 (71)	
	Neutral	6 (17)	
	Disagree	4 (11)	
**The daily messages from Text4Support help me feel like I can bounce back if I make a mistake (n=35)**			
	Agree	20 (57)	
	Neutral	10 (28)	
	Disagree	5 (14)	
**The daily messages from Text4Support help me make better choices (n=35)**			
	Agree	23 (65)	
	Neutral	7 (20)	
	Disagree	5 (14)	
**The daily messages from Text4Support help me improve my overall mental well-being (n=35)**			
	Agree	24 (69)	
	Neutral	5 (14)	
	Disagree	6 (17)	
**The daily messages from Text4Support help me enhance my quality of life (n=35)**			
	Agree	22 (63)	
	Neutral	9 (26)	
	Disagree	4 (11)	

About two-thirds of respondents agreed that the daily TxMs helped them cope with stress (22/35, 63%) and loneliness (23/35, 66%); remember their goals (25/35, 71%); feel hopeful that they could manage issues in their life (22/34, 65%); feel like they know how to stay on track when life or everyday stressors come up (23/35, 66%); feel like they are making a change (25/35, 71%) and are making better choices (23/35, 66%); improve their overall mental well-being (24/35, 69%); and enhance their quality of life (22/35, 63%).

Approximately half of the respondents agreed with the questions related to mood, such as the questions regarding daily texts helping respondents to monitor mood (21/36, 58%); know where to get help for depression or anxiety (16/34, 47%); and feel that they could be in charge of managing depression or anxiety (18/35, 51%).

A total of 57% (20/35) of respondents reported that the daily Text4Support messages helped them feel like they could bounce back upon making a mistake, and only 35% (12/34) of respondents reported that the messages helped them to manage suicidal thoughts.

The results of the chi-square and Fisher exact tests did not show significant difference in reporting on any of the questions related to satisfaction with the Text4Support service based on the respondents’ sex at birth or admitting diagnosis.

### Qualitative Data

#### Overview

**Figure 1:**
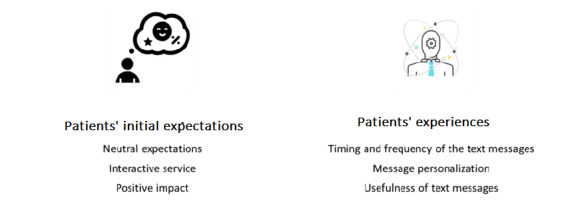
Summative illustration of themes and subthemes.

This aspect of the study was guided by the phenomenological methodological orientation. Thus, we explored how the study candidates make sense of experience with the Text4Support service and transform this experience into a worldview [25]. The study candidates were asked about their own experiences regarding the TxMs they received daily for 6 months. The outcome results were grouped into 2 main themes and 3 further subthemes (Figure 1).

#### Patients’ Initial Expectations of the Program

Before subscription to the Text4Support program, the expectations of the program differed among the study participants. Although some respondents expressed neutrality, not knowing what to expect or whether the program would have any impact on their mental health, others had a positive expectation of their experiences and the impact on their mental health:

I didn’t really know what kind of [supportive] messages they were going to send.

P1

When I heard about [the Text4Support program], I thought, oh, that’ll be good.

P4

One respondent commented that they had expected some form of interactive (2-way flow) component in the messaging program:

Maybe it’s something good because somebody is going to check in with you every day or whatever.

P4

#### Patients’ Experiences With the Program

##### Overview

Patients reported varying experiences with the Text4Support program. Although the program was perceived positively, some respondents were unsure of the impact of the program on their mental health. The reported experiences are categorized into three subthemes as follows: timing and frequency of messages, message personalization, and usefulness of the messages.

##### Timing and Frequency of the TxMs

Generally, participants expressed satisfaction with the timing of the supportive TxMs, which were received daily at noon. For some employed respondents, this timing aligned with their *lunch break* and was perceived as a good opportunity to read and reflect upon the messages:

Well, because [the messages] were [sent] at noon. That was good because you’re usually on lunch break or starting your lunch break, so you don’t get interrupted at work.

P6

The frequency of messages was satisfactory for the patients. The regular and consistent nature of the messages seemed to have improved the perception and impact of the program on the mental health of the patients:

I think that daily message was fine. I think any more would be overwhelming.

P1

In the beginning [the supportive messages] seemed kind of boilerplate, like it wasn’t really something that I could specifically use in my life, [but] as [the program] went on and I got these almost daily messages of techniques and kind of messages of support, I found it very comforting.

P5

##### Message Personalization

Patients expressed their experiences and concerns regarding the personalization of supportive TxMs. Although some patients reported that some of the messages were personally applicable to them, some patients expressed their desire for a more tailored or even interactive program such that it would speak specifically to the patients’ mental health condition or to their particular needs. An interactive program, from the patients’ perspective, would involve or simulate *real* persons communicating back and forth with them:

Some of them [text messages] were very detailed and yes, they applied to me.

P2

They were very generic and very short and very like non-personal. Um, so that part I thought was useless...if you could have a real person doing it, I know they don’t have all day to sit and text you back and forth, but if you had the option to respond, like say they texted, something that was meaningful to your experience or your situation, and then you could interact.

P4

##### Usefulness of TxMs

Patients were very positive about the usefulness and impact of the TxMs on their mental health, and 1 patient commented that using text messaging as a medium was advantageous because they could refer and reflect back on the past TxMs stored on their mobile phones:

If I was having like a day where my anxiety was worse, sometimes the message would be—give me a chance to sit and have time to reflect and be present in that. Um, it also just gave me a chance to think outside of my perception of what I’m experiencing and that too.

P1

Some of [the messages] lifted your spirits or give you direction. They were positive.

P6

Not all patients thought that the messages were helpful. Some commented that the program did not meet their initial expectations. This resulted from the generalized format of some messages. Some believed that more tailored and personalized messages would have been more effective:

I think it varies amongst people and their diagnosis [for which] the messages were being sent. Some were maybe applicable to me, some..., maybe not. So, and this also depends on the perception of the person receiving it.

P1

I was expecting something different other than, well, I got all the text messages, motivational text messages, but I don’t know. I don’t know if it helped me or not.

P6

## Discussion

### Principal Findings

This study sought to understand the experiences of recently discharged acute care patients with mental health concerns and selected to be Text4Support subscribers. The two key goals were as follows: (1) to examine the general experience of Text4Support subscribers and (2) to explore the satisfaction differences based on sex at birth and admitting diagnoses.

The principal findings of the study were that Text4Support was well-perceived; there was a high satisfaction with the messages and the perceived impact of the messages. However, sex at birth and primary diagnosis did not significantly affect satisfaction. Most of the respondents identified as women, had a high education level, were unemployed, were separated or divorced, and were of White ethnicity. These demographic characteristics are common among research respondents who willingly provide feedback to web-based services, including texting messages [2,3,19].

Similar to previous literature, Text4Support had high satisfaction [2,19]. Most subscribers were satisfied and agreed that messages were positive, affirmative, clear, and relevant. In addition, subscribers felt that the messages helped them cope with stress, feel connected to a support system, remember goals, feel hopeful managing life issues, stay on track when life or everyday stressors came up, feel like changes were being made, and make better choices. Subscribers also reported improved mental well-being and enhanced quality of life. In the same context, a systematic review of clinical outcomes from mobile phone and web-based text messaging interventions reported that texting services are praised and well-perceived in the mental health field, adding that texting services have successfully expanded to provide support to diverse psychiatric disorders and during times of crisis [19,26].

This study differs from previous Text4Support papers [19]. The study population included psychiatric patients who were recently discharged from the hospital, whereas previous studies examined individuals who were accessing outpatient services and had a lower severity of mental health concerns.

The period between hospital discharge and the first meeting with a health care provider is perceived as critical and detrimental, and the lack of routine postdischarge follow-up care can lead to early readmission and frequent emergency visits [27-29]. Providing help after discharge through supplementary services, such as supportive TxMs, may help to keep these patients connected with the health care system, especially when the patients are satisfied with the service, and further prevent undesired outcomes. In general, the satisfaction results were consistent with the literature, whereby most texting services reported high satisfaction, a better sense of life control, improved physical health, and increased productivity [30,31], and seemingly regardless of the setting or the type of patient.

Our findings indicate that approximately half of the respondents agreed that daily texts helped them monitor their mood, determine where to get help for depression or anxiety, and feel that they could be in charge of managing their depression or anxiety. This is a lower proportion than that reported by Agyapong et al [2] from subscribers of the Text4Hope program. A lower number of people (compared with Text4Hope subscribers) agreed that texts helped in (1) monitoring mood, (2) determining where to get help, and (3) feeling in charge of managing depression or anxiety. This may be explained by the different type of service provided; although Text4Support was supposed to cater to specific types of mental health concerns (thus their population is more complex and has a mental health concern), Text4Hope was a supportive mental health service provided at a time of crisis (COVID-19 pandemic), and the subscribers were members of the general population. In addition, the COVID-19 pandemic hit at the time of our data collection. This may have imposed excessive psychological burdens, such as stress, anxiety, depression, sleep disorder, and posttraumatic stress disorder symptoms, particularly among those who have underlying mental health conditions [32-34].

One-third of clients self-reported that Text4Support helped them manage suicidal thoughts. In France, a randomized controlled trial using suicide intervention assisted by messages, a supportive text messaging service, was designed to provide communication and support for people contemplating suicide [35]. Initial study results revealed that the intervention was promising as it could maintain communication with patients following discharge from the emergency department, encourage them to contact health care services during crises, and ultimately prevent repeated suicide attempts. This may reflect a critical role of texting services such as Text4Support in providing positive guidance to subscribers during such times of vulnerability. Further research may identify the types of patients who could most benefit from such services during times of crisis.

Regarding the results of the secondary outcome, the relationship of satisfaction with sex at birth or with the primary diagnosis of the participants was not significant. According to the literature, satisfaction based on respondents’ sex at birth or gender usually produces mixed results, although women are more inclined to report to such surveys with positive satisfaction, other research has indicated that men also report high rates of satisfaction [32]. In the same context, patients with different mental health conditions, such as psychotic disorder, depression, anxiety, and comorbid alcohol use, often report high satisfaction with mobile mental health services that make them feel in charge of managing their own mental health symptoms [1,19,31]. These findings may need to be replicated on a larger sample size and at successive time-points to better understand and capture differences based on sex at birth and admitting diagnosis and to track any possible changes that evolve over time.

### Patients’ Expectations Versus Experience

Our results suggest that the initial patients’ expectations were either neutral or positive in relation to the expected nature or the impact of the incoming TxMs on their overall mental health. In addition, the subscribers were satisfied with the frequency of the messages that were provided once daily for 6 consecutive months. Subscribers also recommended and hoped for more personalized messages or mutual interactions with health care personnel. Future services should consider including ways in which clients can personalize their TxMs. The medium of TxMs was perceived as helpful because patients could revisit messages anytime, as they were stored on their mobile phones.

The literature indicates that the majority of patients often expect clinical improvement after a health care intervention, regardless of the service they receive, whereas very few may expect no change or even declining health [18]. It was also reported that patients usually build their expectations in relation to the cost and design or customization of mental health apps, and to a lesser extent put emphasis on the transparency of these apps [36]. Text4Support was a free service for the end users; this may have made it more appealing to complement existing health care services with Text4Support, as combined services are usually preferred by patients in the mental health field [18,19].

Subscribers were satisfied with the frequency of receiving one TxM per day; similar results were obtained from the Text4Mood service [2]. According to one of our subscribers, receiving more frequent messages may be *overwhelming*; however, around 26% (9/34) of participants reported that they preferred the messages to be received twice daily. This discrepancy may highlight the need for more individualized services that can address the patient’s preferences in the services received. In addition, our participants also expressed their satisfaction with receiving the messages at noon, as it aligned with their lunch breaks and provide them with some time to read and reflect on the messages.

Some study participants mentioned that having the TxMs saved on their mobile phones helped enhance and secure the sense of being able to return to the messages anytime. In addition, many liked that they could forward the messages to a friend who may benefit from the service. This finding is in line with those of a similar texting service that found that over 60% of subscribers reported that they returned to the messages at least sometimes [3].

Study subscribers emphasized the importance of individualizing and tailoring TxMs. Furthermore, clients recommended the service to be more interactive, which may be more engaging and supportive. It is usually declared that synchronous programs, where a therapist is involved, can achieve better clinical outcomes and satisfaction among the subscribers [37]. However, these services incur extra costs to the health care system (eg, hiring a clinician or a therapist). Furthermore, therapists may not be available or accessible to support such services; thus, cost-benefit analysis is usually approached under such circumstances. Other options were also available, such as the use of trained volunteers or conversational artificial intelligence systems. A recent study applied a 2-way interactive texting service for patients with chronic medical conditions and their families [38]. Using a design thinking approach, the authors developed a hybrid texting app that allowed the computer to convey bulk messages to the patients, and the health care workers could address them and reply with tailored answers either immediately or within 2 days, according to the urgency.

Generally, asynchronous web-based and text-based services have been accepted by an increasing number of individuals who usually report high satisfaction, ease of use, and better control over life activities (85%), whereas >90% of individuals report increased life productivity after receiving messages from these services [31,39]. In addition, telephone services are frequently associated with a lower attrition rate, compared with face-to-face services, which is likely because of the higher accessibility and lack of geographical barriers [40]. This is also important for those who are hesitant to seek medical attention and may be encouraged to join web-based services [40]. These telephone services could help keep these patients in contact with the health care system.

This study has a number of limitations. First, the small sample size may skew the results and may warrant a larger study that evaluates the service among a larger cohort. In addition, the small sample size means that the study was underpowered, which might affect our ability to detect any differences in satisfaction based on biological sex and primary diagnosis. Second, the messages were partially tailored to the primary diagnosis of the patients; this ratio may need to be increased in future services to fully meet the patients’ requests for TxMs that are personalized to their condition. Third, the results were obtained from 2 different patient groups—those who received TxM alone and those who received TxM in adjunction with PSW; therefore, although it is not highly expected, the PSW may have affected the satisfaction level with the TxM as an outcome of the study. Fourth, we did not compare the quantitative responses with the qualitative responses among the respondents. Finally, although the questionnaire used in the study was designed based on the relevant literature, it was not a validated instrument.

Overall, satisfaction with texting mental health services is well-accepted because texting is convenient, inexpensive, and remotely delivered [3]. According to a systematic review of 27 studies that used mobile phone apps and text messaging, the authors reported that the usability and feasibility along with satisfaction with mobile health services are highly rated by their users [41]. In accordance with this finding, we conclude that Text4Support was well-perceived by the patients who had received the service for 6 months after their discharge from the acute care units. The patients recommended some modifications to the service, including further personalization and interactive services, which may be considered in the design of similar future services.

## Appendix

Multimedia Appendix 1 Key informant telephone interview questions for patients about Text4Support experience.

## Abbreviations

CBT: cognitive behavioral therapy
PSW: peer support worker
TxM: text message
