# Supplementary material for: Patients’ Expectations and Experiences With a Mental Health–Focused Supportive Text Messaging Program: Mixed Methods Evaluation
Source: JMIR Form Res. 2022 Jan 11;6(1):e33438. doi: 10.2196/33438 (PMC8790698; doi:10.2196/33438)
Supplement: Multimedia Appendix 1 [file formative_v6i1e33438_app1.docx]

**Multimedia Appendix 1:**

**Key Informant Telephone Interview Questions for Patients About Text4Support Experience**

1. Can you tell me about your experience with Text4Support?
   1. Have you ever received any type of text messages for mental health before?
   2. Did you like the idea at first (idea of receiving daily texts at first)?
   3. Can you describe what was your expectation before receiving daily texts from Text4Support, did you like the idea?
2. Does receiving daily text messages help you? If so, how do they help you? Please explain.
3. What did you like the best about receiving daily text messages from Text4Support?
4. And what didn’t you like about it?
5. Do you have any challenges with receiving the daily text messages?
6. If so, what are they?
7. What could have been done to help you to overcome this challenge?
8. What are the needs were satisfied when you receive daily text messages?
9. Is it fair to say it is very beneficial to add daily text messages to your treatment as usual plan?
10. Would you recommend daily text messages from Text4Support to family and friends if they need similar support?
11. What were the benefits you gain from receiving daily text messages? Please explain and provide some examples.
12. Do you think that the frequency of receiving daily text messages met your needs?

- If yes, please explain.

- If No, why and what are your suggestions about how these messages can better serve your needs?

1. Would you like to receive daily text messages continuously?
2. From your point of view, how can we improve the daily text messages service?
3. Is there anything else you would like to tell me that you haven’t talked about yet?
